# Effects of the epiphytic patterns on endophytes and metabolites of *Dendrobium nobile* Lindl

**DOI:** 10.3389/fpls.2024.1326998

**Published:** 2024-03-14

**Authors:** Chengxin Yu, Peng Wang, Haiyan Ding, Yuan Hu, Fu Wang, Hongping Chen, Lin Chen, Youping Liu

**Affiliations:** ^1^ School of Pharmacy, Chengdu University of Traditional Chinese Medicine, Chengdu, China; ^2^ State Key Laboratory of Southwestern Chinese Medicine Resource, Chengdu, China

**Keywords:** *Dendrobium nobile* Lindl., epiphytic patterns, metabolites, endophytes, correlation analysis

## Abstract

**Introduction:**

*Dendrobium* is an epiphytic herb plant with neuroprotective, gastroprotective, anti-inflammatory, and immunomodulatory effects. It is often found attached to tree trunks or rocks. With the development of the dendrobium industry, numerous epiphytic patterns exist, such as crushed stone, stump, and sawdust. The study of metabolites and endophytes of *D. nobile* under different epiphytic patterns, which revealed the effects of epiphytic patterns on *D. nobile* from the perspectives of metabolomics and microbiology, is of great significance for the healthy development of *D. nobile*.

**Methods:**

In the study, the *D. nobile* under five epiphytic patterns grown in the same environment were selected. The metabolites were investigated by widely targeted metabolomics, and the endophytes were sequenced using high-throughput sequencing methods. Then, a correlation analysis between the different metabolites and endophytes was performed.

**Results:**

A total of 1,032 metabolites were annotated in *D. nobile*. There are more flavonoids and phenolic acids accumulated on the epiphytic pattern of Danxia stone, whereas the accumulation of lipids on the other epiphytic patterns and 16 differential metabolites was screened out. The endophyte composition of *D. nobile* was dominated by *Proteobacteria*, *Actinomycetes*, *unidentified bacteria, Firmicutes*, and *Cyanobacteria*. For endophytic fungi, *Basidiomycota* and *Ascomycota* were the dominant phyla of *D. nobile*. The relative abundance of *Spirosoma*, *Nocardioides*, and *Arrhenia* in the Danxia stone was significantly higher than that of other epiphytic patterns. According to correlation analysis, we found a significant correlation between differential metabolites and *Spirosoma*, *Nocardioides*, and *Arrheni*.

**Discussion:**

This study confirmed that Dendrobium quality was affected by its epiphytic patterns and revealed its possible causes from a microbiological point of view.

## 1 Introduction

*Dendrobium nobile* Lindl. is a herb that is grown in Southwest China, such as Guizhou, Sichuan, and Guangxi Provinces (Adhikari et al., 2020), and plays a vital role in enhancing health with properties such as neuroprotection (Li et al., 2022a; Li et al., 2022b), anti-inflammation (Lei et al., 2022), antioxidation (Long et al., 2023), and immune modulation (Fan et al., 2020; Long et al., 2023). Studies have shown that *D. nobile* is rich in chemical components such as alkaloids, polysaccharides, phenolic acids, and flavonoids (Ling et al., 2021). In addition, compounds such as sesquiterpenes (Wang et al., 2022a), dibenzyl derivatives (Zhang et al., 2006), and phenanthrene (Cheng et al., 2020) have been isolated from *D. nobile*, which constitute the critical foundation of dendrobium pharmacological quality. In China, *D. nobile* has been used as an ingredient in nutraceutical beverages and food products for thousands of years (Sha and Luo, 1980). Because of its astonishing pharmacological effects and health benefits, Dendrobium tea (Li et al., 2017) and Dendrobium wine (Niu et al., 2020) products have been developed, increasing demand for *D. nobile*.

The quality of traditional Chinese material has always been an essential topic in traditional Chinese medicine research. Previous studies have revealed that cultivation methods and environmental factors influence the quality of traditional Chinese material (Lan et al., 2022; Jia et al., 2023). In the study of the quality of *Dendrobium officinale*, it was confirmed that the pine bark substrate is the optimal cultivation substrate by comparing the differences in metabolites among three substrates: pine bark, coconut coir, and pine bark (Zuo et al., 2020). There is relatively little research on the quality of *D. nobile* with different epiphytic patterns. *D. nobile* is mainly distributed in Guizhou, Sichuan, Guangxi, and other places in China. Among them, the quality of *D. nobile* in Chishui, Guizhou, is the best, and its planting technology is the most advanced (Zhang et al., 2020). Due to its unique Danxia landform, *D. nobile* is mainly cultivated on Danxia stones. With the development of the Dendrobium industry, epiphytic patterns such as crushed stone, sawdust, and stump have emerged (Li et al., 2022c). Therefore, research of the differences in metabolite accumulation under different epiphytic patterns of *D. nobile* is significant for cultivating *D. nobile.*

As an orchid plant, the synthesis and accumulation of secondary metabolites in Dendrobium are regulated by endophytes. An increase in dendrobine content in Dendrobium was reported, and it was found that the fungus MF23 (Mycena sp.) achieved this goal by regulating the expression of genes involved in the mevalonic acid (MVA) pathway (Li et al., 2017). Sujit Shah et al. (Shah et al., 2022) confirmed the production of IAA and metabolites by endophytic fungi isolated from the roots of *Dendrobium longicornu* Lindl and suggested its growth-promoting potential. The correlation between endophytes and metabolites has been revealed in many studies along with the advance of high-throughput sequencing technology. A study revealed significant correlations between differential metabolites and endophytic fungi of *Cynomorium songaricum* Rupr distributed at different locations in China (Shah et al., 2022). Therefore, it is speculated that the epiphytic patterns affect the accumulation of metabolites in *Dendrobium nobile* Lindl by mediating differences in endophyte communities.

Therefore, some questions remain to be understood about the study of *D. nobile*. (i) What are the primary metabolites of *D. nobile* under different epiphytic patterns? (ii) What are the differences in endophytes of *D. nobile* under different epiphytic patterns? (iii) What is the relationship between endophytes and metabolites of *D. nobile* under different epiphytic patterns? To answer these questions, widely targeted metabolomics and high-throughput sequencing methods were used to investigate the metabolites and endophytes of *D. nobile* under different epiphytic patterns. Our results indicated the differences in endophytes and metabolites of *D. nobile* under different epiphytic patterns and suggested the influence of endophytes on the accumulation of metabolites, which provided new insights into the differences in metabolites of *D. nobile* in different epiphytic patterns.

## 2 Materials and methods

### 2.1 Plant material collection and sample preparation

**Figure 1:**
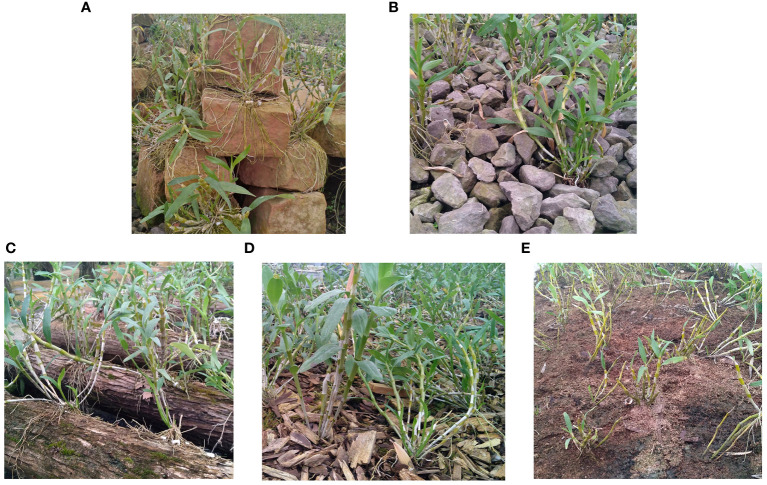
**(A)** The *Dendrobium nobile* Lindl under the epiphytic pattern of DXSK. **(B)** The *Dendrobium nobile* Lindl under the epiphytic pattern of CSS. **(C)** The *Dendrobium nobile* Lindl under the epiphytic pattern of DMZP. **(D)** The *Dendrobium nobile* Lindl under the epiphytic pattern of SPK. **(E)** The *Dendrobium nobile* Lindl under the epiphytic pattern of JMX.

In this experiment, *D. nobile* was collected from Chishui Xintian Company in Zunyi City, Guizhou Province, China, on 15/04/2021. We took the roots and stems of the same plant as the subject of our study. Roots were used to identify the endophytes, and stems were used to separate metabolites. The roots were gently brushed with a sterile brush and placed in new sterile tubes. Sterile phosphate buffer was poured in and vortexed until the sterile phosphate buffer was clarified, quick-frozen in liquid nitrogen, and transferred to a −80°C refrigerator. The stems were frozen with liquid nitrogen and stored in a −80°C refrigerator. The collection epiphytic patterns were (1) Danxia stone (DXSK), crushed stone (CSS), sawdust (JMX): stump (DMZP), and bark block (SPK). Altogether, 30 samples (3 replicates × 5 epiphytic patterns; 15 root samples + 15 stem samples). The five epiphytic patterns are shown in **Figure 1**.

### 2.2 Widely targeted metabolome profiling of *D. nobile*

The fresh stems were freeze-dried by a vacuum freeze-dryer (Scientz-100F), and the freeze-dried samples were crushed for 1.5 min using a mixer mill (MM 400, Retsch) with a zirconia bead. The next steps were as follows: 100 mg of lyophilized powder was dissolved in 1.2 mL of 70% methanol solution and vortexed six times with an interval of 30 min for 30 s each time. Finally, the sample was put in a fridge at 4°C overnight. Then, the extracts were filtered after centrifugation at 12,000 rpm for 10 min (SCAA-104, 0.22-μm pore size); ANPEL, Shanghai, China, http://www.anpel.com.cn/) before UPLC–MS/MS analysis.

A UPLC–ESI–MS/MS system (UPLC, Shimadzu Nexera X2, https://www.shimadzu.com.cn/; MS, Applied Biosystems 4500 QTRAP, https://www.thermofisher.cn/cn/zh/home/brands/applied-biosystems.html) was used in the study. The conditions of analysis were as follows: UPLC: Agilent SB-C18 column (1.8 µm, 2.1 mm * 100 mm). Pure water with 0.1% formic acid (A) and acetonitrile with 0.1% formic acid (B) were used as mobile phases with the following gradient conditions for sample measurements: 0 min, 95% A (5% B); 0 min–9 min, 5% A (95% B); 9 min–10 min, 5% A (95% B); 10 min–11.1 min, 95% A (5% B); and 11.1 min–14 min, 95% A (5% B). The injection volume and the flow velocity were set to 4 μL and 0.35 mL/min, respectively, and the column oven was maintained at 40°C. The effluent was connected to the ESI-triple quadrupole-linear ion trap (QTRAP)-MS.

The triple quadrupole-linear ion trap mass spectrometer (Q TRAP), AB4500 Q TRAP UPLC/MS/MS System, equipped with an ESI Turbo Ion-Spray interface, was used to carry out peak detection, and the program was run in both positive and negative ion modes, controlled by Analyst 1.6.3 software (AB Sciex). The operation parameters of the ESI source were as follows: ion source, turbo spray; source temperature, 550°C; ion spray voltage (IS), 5,500 V (positive ion mode)/−4,500 V (negative ion mode). The pressures of ion source gas I (GSI), gas II (GSII), and curtain gas (CUR) were 50 psi, 60 psi, and 25.0 psi, respectively. The collision-activated dissociation (CAD) was high. In the QQQ and LIT modes, the instrument and calibrated mass were tuned using the 10-μmol/L and 100-μmol/L polypropylene glycol solutions, respectively. To acquire the QQQ scans, the collision gas (nitrogen) was set to medium to perform the MRM experiments. DP and CE were performed on individual MRM transitions and further optimized. A specific set of MRM transitions was monitored during each period based on the metabolites eluted during that period.

### 2.3 Metabolite data processing, statistical analysis, and metabolic pathway analysis

Unsupervised PCA (principal component analysis) and the orthogonal partial least-squares discriminant analysis (OPLSD-DA) were performed by the statistics function prcomp within R (www.r-project.org) and R package MetaboAnalystR, respectively. The steps of OPLS-DA were as follows: log-transformed (log2) and mean-centered of the data were performed before OPLS-DA. The significantly regulated metabolites between groups were screened according to the criteria that VIP ≥1 or absolute log2FC (fold change) ≥1. A permutation test (200 permutations) was performed to avoid overfitting. KEGG annotation and enrichment analysis: The identified metabolites were annotated with the KEGG Compound database (Kanehisa and Goto, 2000) (http://www.kegg.jp/kegg/compound/), and the annotated metabolites were mapped to the KEGG pathway database (http://www.kegg.jp/kegg/pathway.html). Finally, the pathways with significantly regulated metabolites mapped into MSEA (metabolite set enrichment analysis) were input, and their significance was determined by hypergeometric test p values.

### 2.4 DNA extraction and high-throughput sequencing of endophytes

Total genomic DNA was extracted from samples utilizing the CTAB method, and the concentration and purity of the DNA were monitored on 1% agarose gels by diluting the DNA to 1 ng/µL using sterile water according to the concentration. The 16S rRNA/ITS genes of distinct regions (16S V4, ITS1) were amplified using specific primers with the barcodes. All PCR mixtures contained 15 µL of Phusion® High-Fidelity PCR Master Mix (New England Biolabs), 2 µM forward and reverse primers, and approximately 10 ng of template DNA. The steps of thermal cycling were as follows: denaturation at 98°C for 1 min was performed first, and then 30 cycles were followed, including denaturation at 98 °C for 10 s, annealing at 50°C for 30 s, and elongation at 72°C for 30 s. Finally, the samples were incubated at 72°C for 5 min. The PCR products and 1× loading buffer (containing SYBR Green) were mixed with the same volume and detected on a 2% agarose gel electrophoresis. The PCR products were purified with a Qiagen Gel Extraction Kit (Qiagen, Germany) after the PCR products were mixed in equidensity ratios. The sequencing libraries were generated, and the index codes were added using the TruSeq® DNA PCR-Free Sample Preparation Kit (Illumina, USA) with the manufacturer’s recommendations. The quality of the library was assessed on a Qubit@ 2.0 Fluorometer (Thermo Scientific) and Agilent Bioanalyzer 2100 system. The library was sequenced on an Illumina NovaSeq platform, and the paired-end reads with 250 bp were generated.

### 2.5 Statistical analyses

Paired-end reads were assigned to samples based on their unique barcode and truncated by cutting off the barcode and primer sequence. Paired-end reads were merged using FLASH (V1.2.7) (Magoč and Salzberg, 2011). To obtain high-quality clean tags, the raw tags were filtered under specific filtering conditions based on the QIIME (V1.9.1) quality control process. In the condition of the UCHIME algorithm, the detection of the chimera sequences was achieved by comparison between the tags and reference database (Silva database (16S/18S), Unite Database (ITS)) and then removed to derive the effective tags (Caporaso et al., 2010; Bokulich et al., 2013). The sequences were assigned to the same OTUs based on similarity greater than or equal to 97%, and sequence analysis was performed by UPARSE software (Edgar, 2013) (Uparsev7.0. 1001). The representative sequence of each OTU was screened before annotating it. Multiple-sequence alignment was performed using MUSCLE software (Version 3.8.31) to study the phylogenetic relationship of different OTUs and the difference in the dominant species in different samples (groups) (Edgar, 2004). The standard sequence number corresponding to the sample with the fewest sequences was used to normalize the OTU abundance information. The output-normalized data were the foundation of the subsequent alpha and beta diversity analysis. The alpha diversity, a tool to analyze the complexity of species diversity for a sample, was displayed by six indices: Observed-species, Chao1, Shannon, Simpson, ACE, and Good-coverage. The calculation and showing of all the indices in our samples were performed by QIIME (Version 1.7.0) and R software (Version 2.15.3), respectively. The calculation of UniFrac distances was conducted by QIIME software (Version 1.9.1). NMDS plots were plotted using R software (Version 2.15.3). The vegan package of R software was used for NMDS analysis. Beta diversity indices were analyzed for between-group differences using R software, with and without parametric tests; Tukey’s and Wilcox tests of the agricolae package were chosen.

To analyze LEfSe, the LEfSe software was used with a default setting of 4 for the LDA score screening. R software was utilized to perform the Metastats analysis at the phylum, class, order, family, genus, and species levels, to obtain p values from the permutation test between groups, and then ANOSIM, MRPP, and Adonis analyses were conducted utilizing the ANOSIM, mrpp, and Adonis functions of the R vegan package, respectively. AmOVAs were performed using the Mothur software above function. The significant differences between species groups were analyzed using the R software to perform a t test between groups and plotted.

### 2.6 Correlation analysis of dominant genera and differential metabolites

To identify the endophytes that could influence the metabolites, the relationship between endophytes and metabolites of *D. nobile* under five epiphytic patterns was determined by Spearman’s correlation analysis. The coefficients of correlation were marked in colors. Red and blue represented positive and negative correlations, respectively.

## 3 Results

### 3.1 Analysis of metabolite types of *D. nobile*

**Figure 2:**
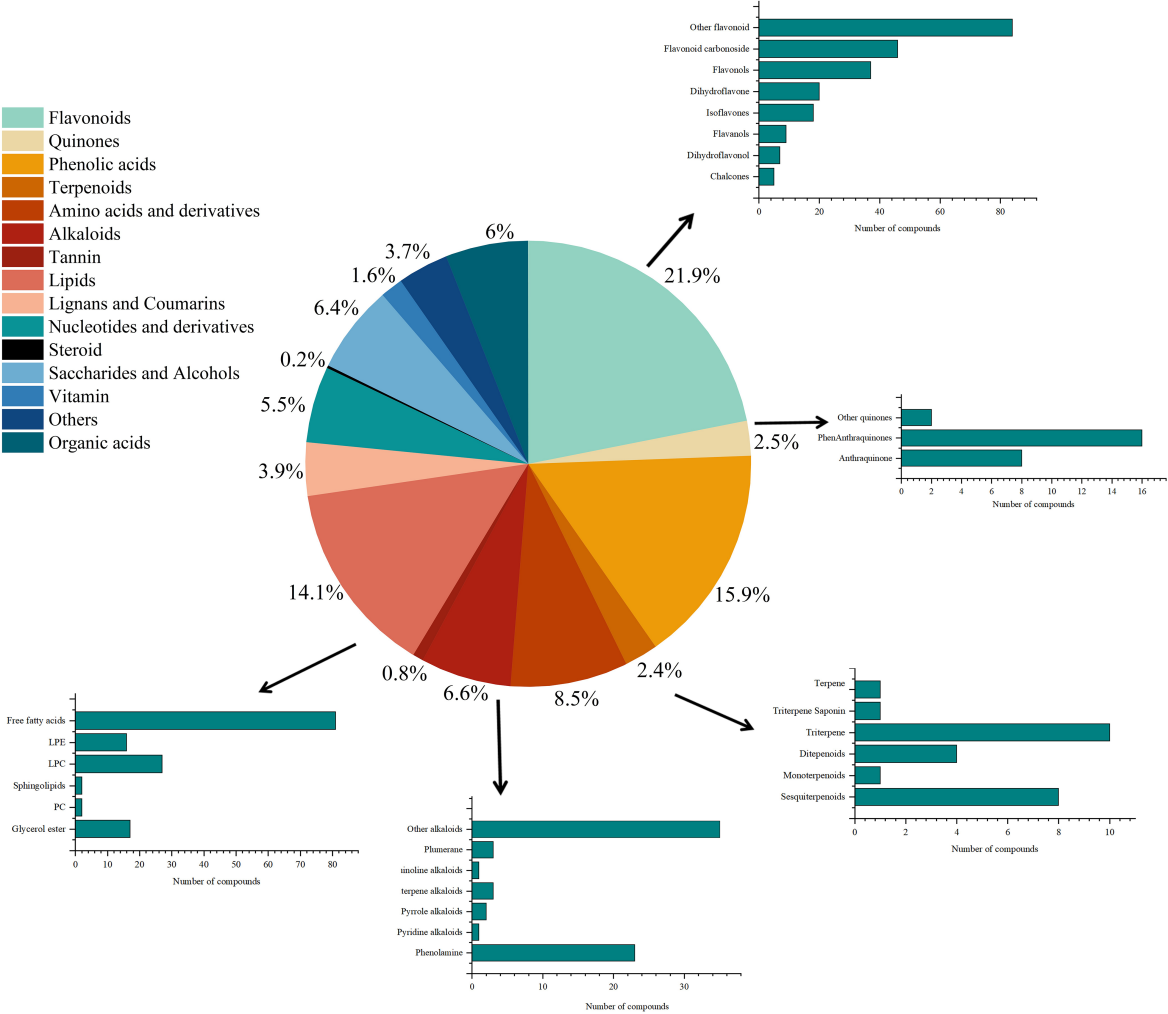
The types and proportions of the metabolites annotated in *D. nobile*.

A total of 1,032 metabolites were annotated in *D. nobile*, including 226 flavonoids, 164 phenolic acids, 145 lipids, 88 amino acids, and their derivatives, 57 nucleotides and their derivatives, 62 organic acids, 66 saccharides and alcohols, 17 vitamins, 68 alkaloids, 40 lignans and coumarins, 26 quinones, 25 terpenoids, 8 tannins, 2 steroids, and 38 other compounds (**Figure 2**). Flavonoid and phenolic acid components accounted for 37.8% of the annotated compounds, as shown in **Figure 1**. The study showed that the flavonoid and phenolic acid were important active components for anti-inflammatory (Ci et al., 2020; Kim et al., 2021; Yuan et al., 2022; Li et al., 2022d), antioxidant, and hypoglycemic effects in Dendrobium. In addition, other alkaloids have been annotated in *D. nobile*, including sesquiterpene alkaloids, pyridine alkaloids, pyrrole alkaloids, plumerane, isoquinoline alkaloids, phenalkamines, and other alkaloids, which were the important basis for the medicinal substances and nutritional quality of *D. nobile*. In addition, primary metabolites such as lipids, amino acids and their derivatives, and organic acids were also detected in *D. nobile*, accounting for 14.1%, 8.5%, and 6% of the total 1,032 metabolites, respectively. It has been reported that the most frequently annotated compounds were flavonoids, phenolic acids, and lipids in *Dendrobium officinale*, with 135, 75, and 72 species, respectively (Zuo et al., 2020), which were in accordance with this study.

### 3.2 Principal component analysis and orthogonal least squares discriminant analysis

**Figure 3:**
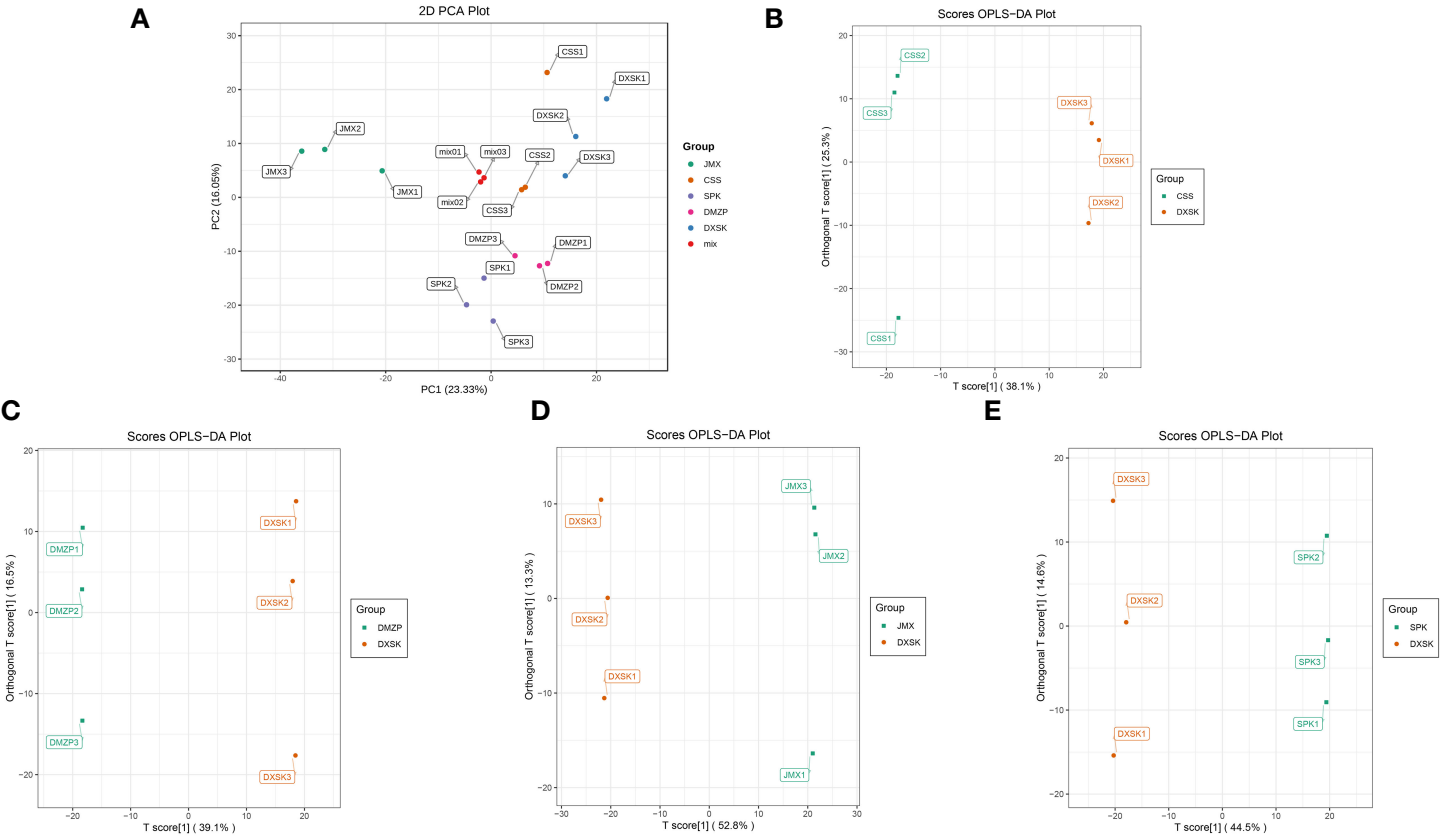
**(A)** The PCA of *D. nobile* samples under five epiphytic patterns. **(B)** The OPLS-DA analysis of *D. nobile* samples between the DXSK and CSS groups. **(C)** The OPLS-DA analysis of *D. nobile* samples between the DXSK and DMZP groups. **(D)** The OPLS-DA analysis of *D. nobile* samples between the DXSK and JMX groups. **(E)** The OPLS-DA analysis of *D. nobile* samples between the DXSK and SPK groups.

The PCA of metabolites in *D. nobile* under different epiphytic patterns was shown in **Figure 3A**: The mixed samples were gathered in a group and separated from other groups, which suggested that the repeatability of mixed samples was good. There was a separation between the JMX group and the other groups in the direction of PC1, and the SPK and DMZP groups were separated in the direction of PC2 compared with the other groups. In addition, the CCS and DXSK groups overlapped greatly not only in the direction of PC1 but also in the direction of PC2. The overall results suggested that the samples of DXSK and CSS were different from the samples of SPK, JMX, and DMZP. The OPLS-DA analysis of the DXSK group and other groups was carried out, and the results are shown in **Figures 3B–E**); the samples of the DXSK group with other groups had a significant degree of separation, which indicated that there were differences in the metabolites of *D. nobile* under different epiphytic patterns. Moreover, the model predictive parameters Q^2^ and R^2^Y of CSS vs. DXSK, DMZP vs. DXSK, JMX vs. DXSK, and SPK vs. DXSK were 0.79 and 0.999, 0.838 and 1, 0.95 and 1, and 0.913 and 0.998, respectively, which indicated that the model was stable and reliable (**Supplementary Figure 1**).

### 3.3 Identification of differential metabolites of *D. nobile* under five epiphytic patterns

**Figure 4:**
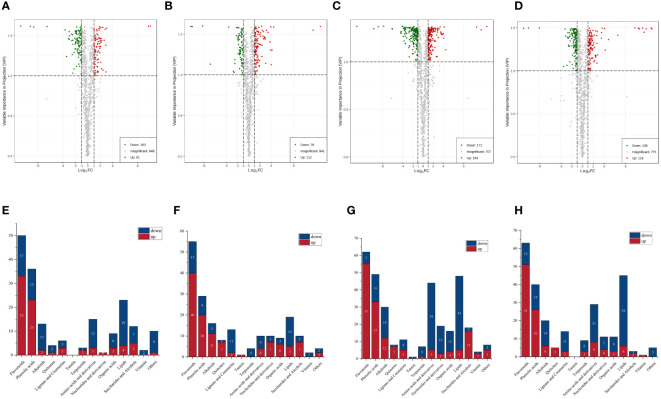
**(A)** Volcano plots for CSS-DXSK. **(B)** Volcano plots for DMZP-DXSK. **(C)** Volcano plots for JMX-DXSK. **(D)** Volcano plots for SPK-DXSK (the green dots in the plots illustrated that the differential metabolites were significant and downregulated, whereas the red dots illustrated that the differential metabolites were significant but upregulated, and the black dots illustrated that the metabolites could be detected in samples but did not have any significant difference). **(E)** The quantities of up- and downregulated metabolites between the CSS and DXSK groups. **(F)** The quantities of up- and downregulated metabolites between the DMZP and DXSK groups. **(G)** The quantities of up- and downregulated metabolites between the JMX and DXSK groups. **(H)** The quantities of up- and downregulated metabolites between the SPK and DXSK groups (red represents the number of metabolites with significantly increased relative content in the latter group, whereas dark blue represents the number of metabolites with significantly decreased content in the latter group).

Differential metabolite screening was performed using fold change values from univariate analysis and variable importance in project (VIP) values from orthogonal partial least squares discriminant analysis models for multivariate analysis. From **Figure 4A**, it can be seen that there were 184 metabolites with significantly different contents between the CSS and DXSK groups. Compared with CSS, DXSK contained 81 metabolites with an upregulated state and 103 metabolites with a downregulated state. A total of 190 metabolites were significantly different in content between the DMZP and DXSK groups (**Figure 4B**), among which 112 metabolites increased and 78 metabolites decreased in content in the DXSK group compared with the DMZP group. There were 325 metabolites recognized as significantly different between JMX and DXSK (**Figure 4C**). Among these metabolites, there were 154 metabolites with significantly higher content in DSXK than in JMX. A total of 256 metabolites were significantly different in content between the SPK and DXSK groups (**Figure 4D**); 118 metabolites increased, and 138 metabolites decreased in content in the DXSK group compared with the SPK group.

**Figure 5:**
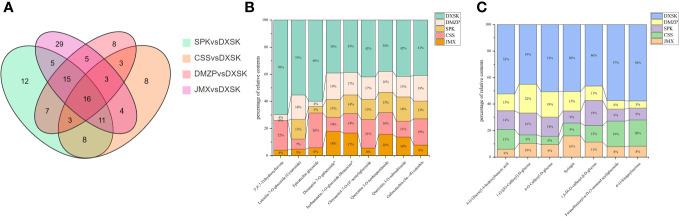
**(A)** Venn diagram illustrating shared or unique metabolites that differed significantly in terms of content among the different comparisons. **(B)** Percentage of relative content of nine flavonoids in different groups. **(C)** Percentage of relative content of seven phenolic acids in different groups.

To analyze the differences of metabolites under different epiphytic patterns of *D. nobile* more clearly. The metabolites were divided into 14 groups. As shown in **Figure 4E**, compared with CSS, there were 33 out of 50 flavonoids upregulated and 23 out of 33 phenolic acids upregulated in DXSK; the contents of 40 flavonoids and 20 phenolic acids were increased significantly in DXSK compared with DMZP (**Figure 4F**); compared with JMX, there were 55 out of 62 flavonoids upregulated and 33 out of 49 phenolic acids upregulated in DXSK(**Figure 4G**), and the contents of 51 flavonoids and 26 phenolic acids were increased significantly in DXSK compared with SPK (**Figure 4H**). Furthermore, the distribution of all metabolites that differed significantly in content showed that there were also large fluctuations in the content of lipids (**Supplementary Table 1**), as shown in **Figures 4E–H**, compared with the DXSK group, the contents of most lipids increased significantly in CSS, DMZP, JMX, and SPK. Upon further screening of flavonoids and phenolic acids that were significantly upregulated in DXSK, as shown in **Figure 5A**, 16 metabolites were screened, and their contents were significantly increased in DXSK (**Figures 5B, C**). The metabolite information is shown in **Supplementary Table 2**.

**Figure 6:**
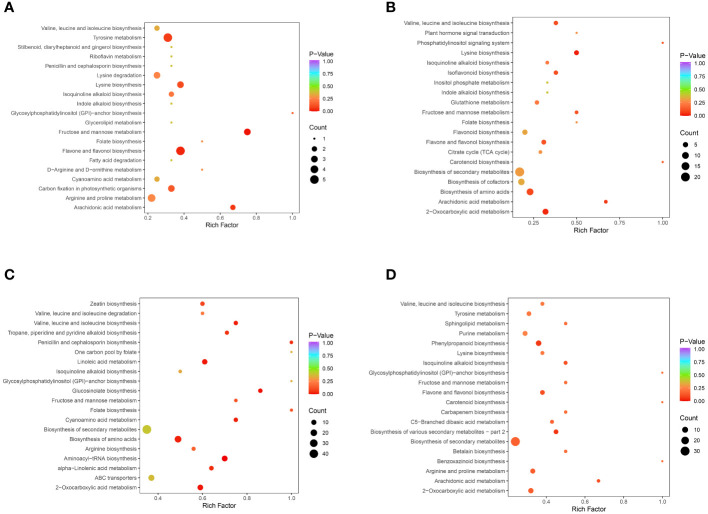
**(A)** KEGG enrichment maps of differential metabolites between CSS and DXSK. **(B)** KEGG enrichment maps of differential metabolites between DMZP and DXSK. **(C)** KEGG enrichment maps of differential metabolites between JMX and DXSK. **(D)** KEGG enrichment maps of differential metabolites between SPK and DXSK (the abscissa represents the enrichment factor of the pathway, and the ordinate shows the names of pathways. The color of the dot represents the p-value, and the deeper the red of the dot, the stronger the enrichment effects. The size of points represents the number of metabolites enriched in the pathways).

KEGG pathway analysis was performed to integrate genes, expression, and metabolites into a complete network for study, showing a higher level of biological function. Differential metabolites were mapped to the KEGG database, and annotation results for important metabolites were ranked according to pathway type. The results of the KEGG pathway enrichment analysis are shown in **Figures 6A–D**. The differential metabolites in the comparisons of CSS vs. DXSK were annotated in 20 pathways, among which flavonoid and flavonol biosynthesis and fructose and mannose metabolism changed obviously (*P*<0.05). Five and three differential metabolites were involved in these two pathways, respectively. Similarly, the differential metabolites between DMZP and DXSK were annotated in 20 pathways, and the lysine biosynthesis and 2-oxocarboxylic acid metabolism altered prominently (*P*<0.05). There were four and seven differential metabolites involved in these two pathways, respectively. The differential metabolites between JMX and DXSK were involved in 20 pathways. The pathways, including aminoacyl-tRNA biosynthesis and glucosinolate biosynthesis, changed significantly (*P*<0.01). There were 14 and 6 differential metabolites involved in these two pathways, respectively.

### 3.4 Analysis of the diversity of endophytes of *D. nobile* under five epiphytic patterns

**Figure 7:**
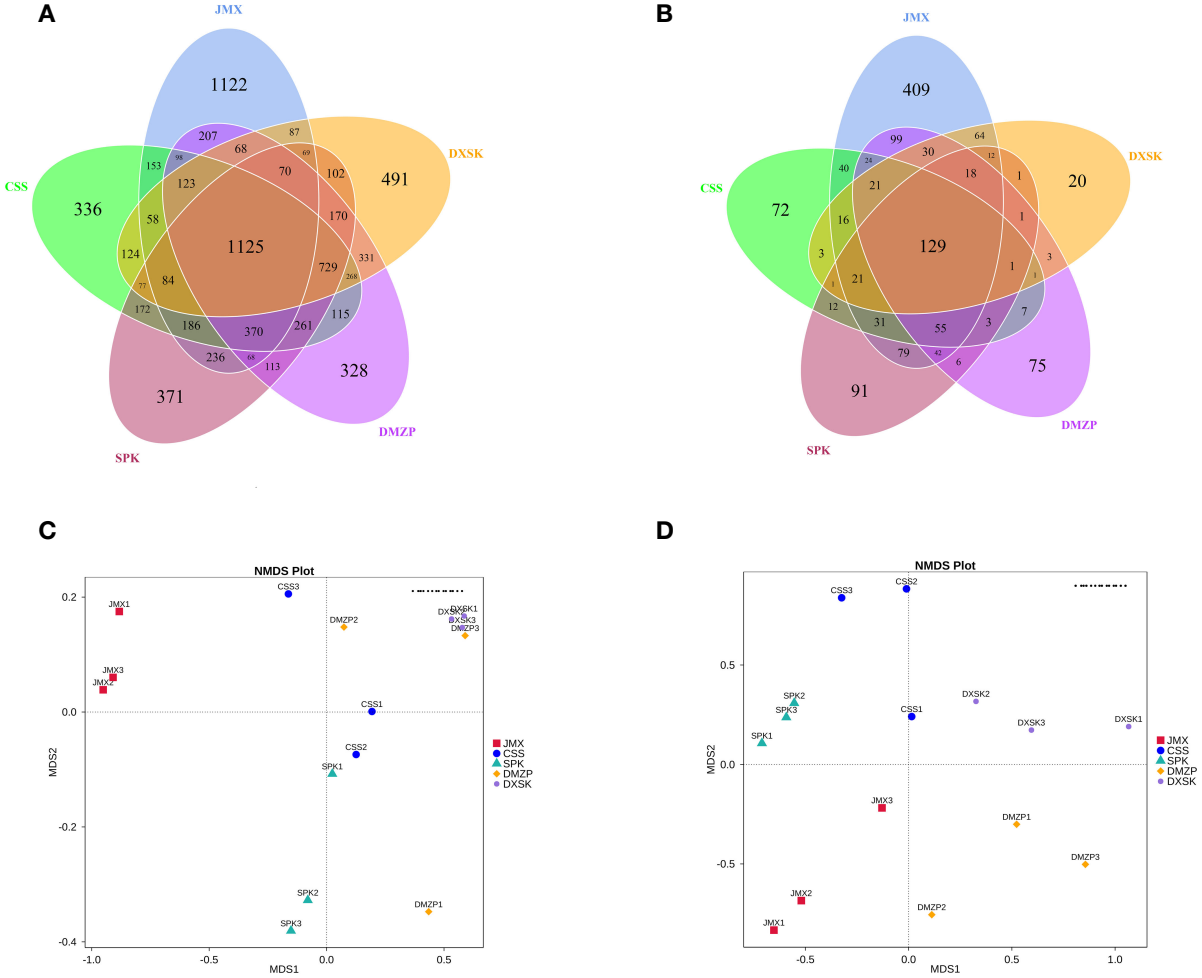
**(A)** The Venn diagrams of the number of OTUs of endophytic bacteria in different groups. **(B)** The Venn diagrams of the number of OTUs of endophytic fungi in different groups. **(C)** The NMDS plot of endophytic bacteria in different groups. **(D)** The NMDS plot of endophytic fungi in different groups.

The DNA sequences of *D. nobile* were analyzed by Illumina-MiSeq sequencing technology; an average of 68,237.33, 61,125.67, 60,112.00, 62,744.67, 60,513.67 high-quality sequences of endophytic bacteria and 84,289.00, 74,150.67, 82,171.67, 79,404.00, and 62,699.67 high-quality sequences of endophytic fungi were obtained from the DXSK, CSS, DMZP, JMX, and SPK groups, respectively (**Table 1**). After clustering the samples, a Venn plot was drawn. From **Figures 7A**, **B**, there were differences in the number of endophytic OTUs in different groups. The number of OTUs of endophytic bacteria was the highest in DMZP and the number of OTUs of endophytic fungi was the highest in SPK. Furthermore, the proportion of OTUs of endophytes in DXSK was relatively small compared with other groups. The proportion of OTUs in common was relatively low among the five groups, with endophytic bacteria ranging from 25.3% to 33.3% and the endophytic fungi ranging from 11.8% to 37.7%, indicating significant differences in the composition of endophyte populations in *D. nobile* under different epiphytic patterns.

**Table 1:** Alpha diversity index of endophytes in different groups.

Group	Endophytic bacteria			Endophytic fungi		
	Sequence	Shannon	Chao1	Sequence	Shannon	chao1
JMX	62,744.67 ± 1034.54	8.89 ± 0.26	2,856.91 ± 120.37	79,404.00 ± 9075.13	4.95 ± 0.41^a^	785.19 ± 87.85^a^
SPK	60,513.67 ± 3602.60	8.15 ± 0.21	2,922.84 ± 145.32	62,699.67 ± 16263.56	1.70 ± 0.19	394.48 ± 85.31^b^
DMZP	60,112.00 ± 8878.46	8.40 ± 0.83	2,823.48 ± 510.88	82,171.67 ± 2736.20	3.82 ± 0.91^ab^	335.48 ± 85.31^b^
CSS	61,125.67 ± 1305.13	7.99 ± 0.74	2,991.14 ± 187.58	74,150.67 ± 12914.54	2.90 ± 1.22^b^	318.61 ± 66.81^b^
DXSK	68,237.33 ± 1130.72	8.93 ± 0.25	2,858.49 ± 189.63	84,289.00 ± 3414.14	3.18 ± 0.72^b^	254.02 ± 60.77^b^

Different letters represent significant differences.

The Shannon and Chao1 indices were used to assess the alpha diversity of the endophytic community. The endophytic bacterial diversity indices of the five groups had no significant difference; for endophytic fungal diversity, the Shannon index and Chao1 index of JMX were higher than in other groups, and the Chao1 index of DXSK was lower than in others (**Table 1**).

Through NMDS analysis, classification of multiple samples can be achieved, further demonstrating differences in species diversity among samples. The more similar the composition of microbial communities in the samples, the closer they are on the coordinate map. Regarding endophytic bacteria, the samples from JMX and DXSK groups were separated in the direction of MDS1, and those from SPK and DXSK were separated in the direction of MDS2. The samples from CSS and DMZP were relatively close to the samples of DXSK in both directions, MDS1 and MDS2. Regarding endophytic fungi, the samples from SPK and DXSK groups were separated in the direction of MDS1, and those from JMX and DXSK were separated in the direction of MSD1 and MDS2. The samples from CSS and DMZP were relatively close to the samples of DXSK in the direction of MDS1 (**Figures 7C, D**).

### 3.5 Distribution and diversity of endophytes of *D. nobile* under different epiphytic patterns

**Figure 8:**
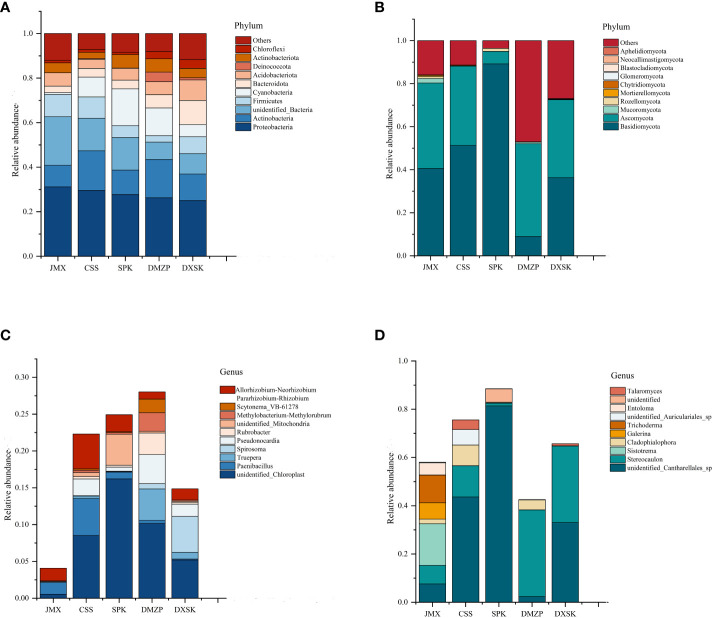
**(A)** Composition and relative abundance of endophytic bacterial in different groups on phylum level; **(B)** Composition and relative abundance of endophytic fungal in different groups on phylum level; **(C)** Composition and relative abundance of endophytic bacterial in different groups on genus level; **(D)** Composition and relative abundance of endophytic fungal in different groups on the genus level.

The annotated phyla and genera were statistically analyzed to understand the differences in microbial composition between *D. nobile* under different epiphytic patterns (**Figure 8**). The top 10 endophytic bacteria with relative abundance at the phylum level were *Proteobacteria, Actinobacteria, Unidentified_Bacteria, Firmicutes, Cyanobacteria, Bacteroidota, Acidobacteriota, Deinococcota, Actinobacteriota*, and *Chloroflexi*. Among them, five endophytic bacteria in all groups, namely, *Proteobacteria, Actinobacteria, Unidentified_Bacteria, Firmicutes*, and *Cyanobacteria*, were dominant phyla, with the sum of relative abundance accounting for more than 70% of the total relative abundance of all bacterial phyla. The relative abundances of *Bacteroidota* (10.82%) and *Chloroflexi* (3.96%) in the DXSK were significantly higher than in other groups; the relative abundances of Cyanobacteria in the SPK and DMZP were significantly higher than in other groups, with relative abundances of 16.54% and 12.44%, respectively. For endophytic fungi, the top 10 endophytic fungi with relative abundance at the phylum level were *Basidiomycota, Ascomycota, Mucoromycota, Rozellomycota, Mortierellomycota, Chytridiomycota, Glomeromycota, Blastocladiomycota, Neocallimastigomycota*, and *Aphelidiomycota* in all groups. The *Basidiomycota* and *Ascomycota* were the dominant phyla, with the sum of relative abundance accounting for more than 70% of the total relative abundance of all bacterial phyla. Compared with the other four groups, the SPK had a higher relative abundance of *Basidiomycota* and *Rozellomycota* and a lower relative abundance of *Ascomycota. Mortierellomycota* predominated in JMX, and Basidiomycota relative abundance was lowest in the group of DMZP.

At the genus level, the distribution of the top 10 bacterial genera in relative abundance among all groups is shown in **Figures 8C, D**. The dominant endophytic bacterial genera were mainly *unidentified_Chloroplast, Paenibacillus, Truepera, Spirosoma, Pseudonocardia, Rubrobacter, unidentified-Mitochondria, Methylobacterium–Methylorubrum, Scytonema-VB-61278, Allorhizobium–Neorhizobium–Parararhizobium–Rhizobium*. Compared with other groups, the relative abundances of *Truepera, Pseudonocardia*, and *Rubrobacter* in the DMZP were all higher, and the relative abundance of *Spirosoma* in DXSK was higher. For endophytic fungi, the dominant endophytic fungal genera were mainly *unidentified_ Cantharellales_sp, Stereocaulon, Sistotrema, Cladophialophora, Galerina, Trichoderma, unidentified_Auriculariales_sp, Entoloma*, and *Talaromyces*. Among them, the relative abundances of Sistotrema and Trichoderma in the JMX were higher than in other groups.

### 3.6 Analysis of the specific endophytes of *D. nobile* under different epiphytic patterns

**Figure 9:**
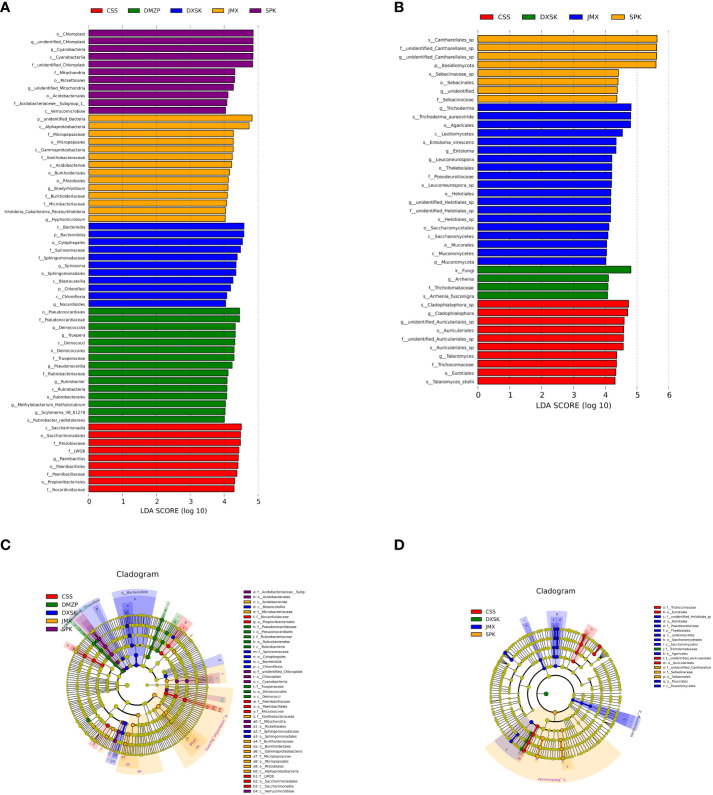
LEfSe analysis of endophyte communities of different groups (**A, C**: endophytic bacteria; **B, D**: endophytic fungi).

To investigate whether species with significant differences in Dendrobium endophytes under different epiphytic patterns, the LEfSe analysis was used. We detected significant differences in the abundance of endophyte biomarkers from different groups. As shown in **Figure 9**, the absolute value of the LDA score in the figure represents the magnitude of the effect of the differential species; in the JMX, the significantly abundant taxa were the genera *Bradyrhizobium, burkholderia_Caballeronia_Paraburkholderia*, and *Hyphomicrobium* and the families *Micropepsaceae*, *Xanthobacteraceae, Burkholderiaceae*, and *Microbacteriaceae*; it has been reported that *Bradyrhizobium* fixes nitrogen, which can fix atmospheric nitrogen to combined nitrogen that is used by the host plant (Liu, 2019), and *Hyphomicrobium* are denitrifying, which are harmful bacteria (Li et al., 2019). In the CSS, the significantly abundant taxa were the genera f_LWQ8 and *Paenibacillus* and the families *Rhizobiaceae, Paenibacillaceae*, and *Nocardioidaceae*. A study found that *Paenibacillus* can not only secrete *indorioacetate* or gibberellin, thus effectively promoting the growth of plants, but also can inhibit the activity of a variety of pathogens, thus significantly improving the disease resistance of plants (Li et al., 2014). In the SPK, the significantly abundant taxa were the genus *unidentified_Mitochondria, Acidobacteriaceae_Subgroup_1*, *unidentified_Chloroplast*, and *unidentified_Mitochondria.* In the DMZP, the significantly abundant taxa were the genera Truepera, *Pseudonocardia, Rubrobacter, Methylobacterium-methylorubrum*, and *Scytonema_VB_61278* and the family *Rubrobacteriaceae.* The existing studies show that some isolated strains of *Pseudonocardia* show nitrogen fixing and can perform heterotrophic ammonia oxidation (Liu et al., 2005), and the genus *Methylobacterium-methylorubrum* has a good nitrogen-fixing capacity (Zhao, 2016). In the DXSK, the significantly abundant taxa were the genera *Spirosoma* and *Nocardioides* and the families *Spirosomaceae* and *Sphingomonadaceae.* The *Nocardioides* is a typical genus of *Noucaceae (Nocardioidaceae)* of *Actinobacteria* (*Actinobacteria*), with a certain salt tolerance, and can use a variety of organic compounds as the only carbon source (Du et al., 2012). Some strains have catabolism of a series of *methiontriazine* herbicides and other organic compounds (Hamamura and Arp, 2000; Kubota et al., 2005) and have a certain inhibitory ability.

In terms of endophytic fungi. In the JMX, the significantly abundant taxa were the genera unidentified *Helotiales_sp*, *Trichoderma*, *Entoloma*, and *Leuconeurospora* and the family *Pseudeurotiaceae*. In the CSS, the significantly abundant taxa were the genera *unidentified_Auriculariales_sp*, *Cladophialophora*, *Talaromyces*, and *Cladophialophora_sp* and the family *Trichocomaceae.* In the SPK, the significantly abundant taxa were the genus *unidentified_Cantharellales_sp*, the family *unidentified_Cantharellales_sp*, and *Sebacinaceae.* In the DXSK, the significantly abundant taxa were the genus *Arrhenia* and the family *Tricholomataceae.*

### 3.7 The relationship between endophytes and differential metabolites

Elucidating the relationship between endophytes and their metabolites in *D. nobile* under different substrates is important for *D. nobile* research. In this study, the correlation between microorganisms and metabolites was analyzed according to relative abundance and content.

**Figure 10:**
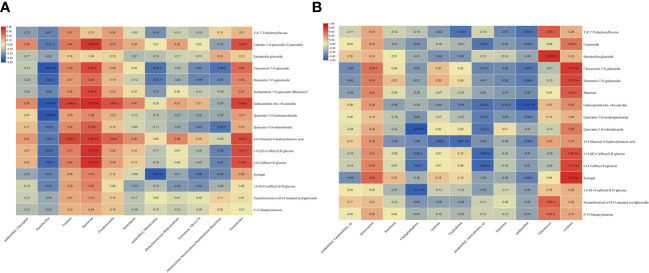
**(A)** Heatmap of the correlation between endophytic bacteria and differential metabolites. **(B)** Heatmap of the correlation between endophytic fungi and differential metabolites (red represents a positive correlation, blue indicates a negative correlation, and the darker the color, the stronger the correlation) * *P* <0.05; ** *P*<0.01.

The correlation between the endophytes and the 16 metabolites in DXSK was investigated. In endophytic bacteria, *Paenibacillus* was associated with cynaroside, chrysoeriol-7-O-glucoside, diosmetin-7-O-galactoside, gallocatechin-(4α→8)-catechin, quercetin-3-O-neohesperidoside, quercetin-3-O-robinobioside, 4-O-glucosyl-4-hydroxybenzoic acid, 1-O-[(E)-caffeoyl]-D-glucose, and 6-O-caffeoyl-D-glucose were significantly negatively correlated; *Truepera* and *Pseudonocardia* were significantly positively correlated with gallocatechin-(4α→8)-catechin and 4-O-glucosyl-4-hydroxybenzoic acid; *Spirosoma* was significantly positively correlated with cynaroside, chrysoeriol-7-O-glucoside, brassicin, gallocatechin-(4α→8)-catechin, quercetin-3-O-robinobioside, 4-O-glucosyl-4-hydroxybenzoic acid, and 1-O-[(E)-caffeoyl]-D-glucose, 6-O-caffeoyl-D-glucose. *Unidentified_Mitochondria* was significantly correlated with chrysoeriol-7-O-glucoside; diosmetin-7-O galactoside and syringin were significantly negatively correlated; *Nocardioides* were significantly correlated with cynaroside, gallocatechin-(4α→8)-catechin, 4-O-glucosyl-4-hydroxybenzoic acid, 1-O-[(E)-caffeoyl]-D-glucose, and 6-O-caffeoyl-D-glucose (**Figure 10A**).

Among endophytic fungi, *Stereocaulon* was significantly and positively correlated with chrysoeriol-7-O-glucoside and syringin; *Cladophialophora* was significantly and negatively correlated with quercetin-3-O-robinobioside; 1-O-[(E)-caffeoyl]-D-glucose, 6-O-caffeoyl-D-glucose, and 1,6-di-O-caffeoyl-β-D-glucose were significantly negatively correlated; Trichoderma was negatively correlated with 3′,4′,7-trihydroxyflavone and 4-O-Glucosyl-4 hydroxybenzoic acid; *unidentified_Auriculariales_sp* was significantly negatively correlated with cynaroside, and chrysoeriol-7-O-glucoside, gallocatechin-(4α→8)-catechin, quercetin-3-O-neohesperidoside, quercetin-3-O-robinobioside, 1-O-[(E)-caffeoyl]-D-glucose, and 6-O-caffeoyl-D-glucose were significantly negatively correlated; *Entoloma* was negatively correlated with gallocatechin-(4α→8)-catechin; *Talaromyces* was negatively correlated with 3′,4′,7-trihydroxyflavone, and epicatechin glucoside, furanofructosyl-α-D-(3-mustard acyl) glucoside, 6′-O-sinapoylsucrose were significantly positively correlated; *Arrhenia* was significantly correlated with cynaroside, chrysoeriol-7-O-glucoside, diosmetin-7-O-galactoside, brassicin, 1-O-[(E)-caffeoyl]-D-glucose, and 6-O-caffeoyl-D-glucose, and syringin showed significant positive correlations (**Figure 10B**).

## 4 Discussion

In the past, studies on *D. nobile* focused on the active ingredients and their pharmacological activities, and the ingredients mainly included alkaloids, polysaccharides, and benzyls (Ling et al., 2021; Wu et al., 2023). There are a few studies on the quality control of Dendrobium medicinal materials and its influencing factors.

In this study, the differences in metabolites of *D. nobile* under different epiphytic patterns were determined by widely targeted analysis, and the results showed that the different epiphytic patterns led to different metabolite accumulation in Dendrobium. Compared with other patterns, more flavonoids and phenolic acids are accumulated on the epiphytic pattern of Danxia stone. We found 16 metabolites, including 3′,4′,7-trihydroxyflavone, lignanin-7-O-glucoside (lignanoside), epicatechinoside, gold sage flavin-7-O-glucoside, geranoside 7-O-galactoside, isorhamnetin-7-O-glucoside (cranberry glycoside), gallocatechin-(4α→8)-catechin, quercetin 3-O-neo-orange peel glucoside, quercetin 3-O-acanthoside, 4-O-glucosyl-4-hydroxybenzoic acid, 1-O-[(E)-caffeoyl]-D-glucose, 6-O-caffeoyl-D-glucose, lilacoside, 1,6-bis-O-caffeoyl-β-D-glucose, furanosyl-α-D-(3-mustardyl) glucoside, and 6′-O-mustardyl sucrose whose contents were significantly increased under the epiphytic pattern of Danxia stone. In our study, these 16 metabolites more objectively reflected the metabolically different epiphytic patterns, although they were not identified as traditional compounds, which would be able to evaluate the quality of *D. nobile*.

Furthermore, the similarities and differences of the endophytes of *D. nobile* under five epiphytic patterns were analyzed, which provided a vital foundation for understanding the relationship between endophytes and the diversity and composition of metabolites. Microbial community diversity is closely related to soil properties (Tkacz et al., 2015) and the physicochemical properties of cultivation substrates, such as pH and elemental content (Cao et al., 2021). For example, soil type was found to be an important factor influencing the composition of rhizosphere and endophytes in *Arabidopsis thaliana* (Bulgarelli et al., 2012). In this study, we found that the α-diversity of endophytic fungi in JMX was significantly higher than that of other groups, whereas the chao1 index of DXSK was significantly lower than that of the rest of the groups. The occurrence of these differences may be related to the different physicochemical properties of the epiphytic substrates, but further studies are needed to verify this. Furthermore, we found that the endophytic bacteria including *Proteobacteria, Actinomycetes, unidentified bacteria, Firmicutes* and *Cyanobacteria* were the dominant bacterial phyla, and the *Amoebacteria, Actinomycetes, thick-walled Bacteria*, and *Cyanobacteria* are the common endophytic bacterial phyla of Orchidaceae (Tsavkelova et al., 2007; White et al., 2014). For endophytic fungi, *Basidiomycota* and *Ascomycota* were the dominant fungal genera. Our group’s previous research on the diversity of the rhizosphere of Dendrobium in different epiphytic substrates showed that the dominant bacteria of Dendrobium are *Proteobacteria, Firmicutes, Bacteroidota*, and *Actinobacteriota*; the dominant fungi are mainly the phyla *Basidiomycota* and *Ascomycota.* The results of this study are similar to these results, indicating that the endophyte community of Dendrobium is influenced by the environment and that there is a correlation between it and the rhizosphere (Wang et al., 2023).

Previous studies have shown that endophytes can promote the production of secondary plant metabolites or produce their metabolites of medicinal value (Cui et al., 2018; Zhou et al., 2020; Li et al., 2021; You et al., 2021). We first found that the metabolites of Dendrobium correlated with endophytes, which provided a foundation for endophytes and chemical studies. In our study, endophytes were found to be differentially correlated with the differential metabolites by correlation analysis, with the endophytic bacterial genera *Spirosoma* and *Nocardioides*, which were significantly enriched in *D. nobile* under the Danxia stone substrate, correlating with lignan, gallocatechin-(4α→8)-catechin, 4-O-glucosyl-4-hydroxybenzoic acid, 1-O-[(E)-caffeoyl]-D-glucose, and 6-O caffeoyl-D-glucose, which were significantly and positively correlated. The endophytic fungus Arrhenia was significantly and positively correlated with lignan, gold sage flavin-7-O-glucoside, vanilloid-7-O-galactoside, tracheloside, 1-O-[(E)-caffeoyl]-D-glucose, 6-O-caffeoyl-D-glucose, and lilacin, suggesting that compared with other substrates, the tansy substrate was significantly correlated. The study found that lignan has antioxidant, anti-inflammatory, and anticancer (Yu et al., 2019) effects, but there has been no report in Dendrobium, and lilacin has anticancer, antioxidant, and immunomodulatory effects (Gong et al., 2014), which have been reported in Dendrobium (Wang et al., 2019). Previous studies have confirmed that the Nocardioides bacterium isolated from the soil of a ginseng field was determined to have β-glucosidase activity and the ability to transform ginsenoside Rb1 (one of the dominant active components of ginseng) to F2 via gypenoside XVII and Rd (Kim et al., 2013). In addition, positive correlations have also been found between *Nocardioides* and the volatile components such as styrene, β-pinene, β-myrcene, and caryophyllene (Wang et al., 2022b), suggesting that *Nocardioides* were involved in the biosynthesis and accumulation of certain metabolites, but the specific role needs to be further validated.

## 5 Conclusion

This study confirmed that Dendrobium quality was affected by the epiphytic patterns and revealed its possible causes from a microbiological point of view. Compared with other epiphytic patterns, Danxia stone was more favorable for the accumulation of flavonoids and phenolic acids in *D. nobile*, and 16 differential metabolites were screened out in *D. nobile* between Danxia stone and other epiphytic patterns. There were differences in the community structure and relative abundance of endophytes of *D. nobile* under the five epiphytic patterns, and the specific flora was different. Furthermore, there was a certain correlation between the content of screened differential metabolites and the relative abundance of endophytes, which revealed that epiphytic patterns mediated the differences of endophytes and thus affected the differential synthesis and accumulation of metabolites.

## Data availability statement

The datasets presented in this study can be found in online repositories. The names of the repository/repositories and accession number(s) can be found below: https://www.ncbi.nlm.nih.gov/, PRJNA1010465.

## Author contributions

CY: Data curation, Formal analysis, Writing – original draft. PW: Data curation, Formal analysis, Writing – original draft. HD: Data curation, Formal analysis, Writing – original draft. YH: Investigation, Writing – original draft. FW: Investigation, Writing – original draft. HC: Writing – original draft. LC: Writing – original draft. YL: Writing – review & editing.
