# Supplementary material for: Effects of the epiphytic patterns on endophytes and metabolites of Dendrobium nobile Lindl
Source: Front Plant Sci. 2024 Mar 14;15:1326998. doi: 10.3389/fpls.2024.1326998 (PMC10972854; doi:10.3389/fpls.2024.1326998)
Supplement: Supplementary file 1 [file DataSheet_1.docx]

Supplementary Material

Effects of the Epiphytic patterns on endophytes and metabolites of *Dendrobium nobile* Lindl

Chengxin Yu^1,2^, Peng Wang^1,2^, Haiyan Ding^1,2^, Yuan Hu^1,2^, Fu Wang^1,2^, Hongping Chen^1,2^, Lin Chen^1,2^* and Youping Liu^1,2^*

1 Department of Pharmacy, Chengdu University of Traditional Chinese Medicine, Standardization Education Ministry Key Laboratory of Traditional Chinese Medicine, Sichuan 611137, China

2 State Key Laboratory of Southwestern Chinese Medicine Resource, Sichuan 611137, China

*** Correspondence:** Lin Chen: [chenlin@cdutcm.edu.c;](mailto:chenlin@cdutcm.edu.c;) Youping Liu: youpingliu@cdutcm.edu.cn

# Supplementary Figures and Tables

## Supplementary Figures


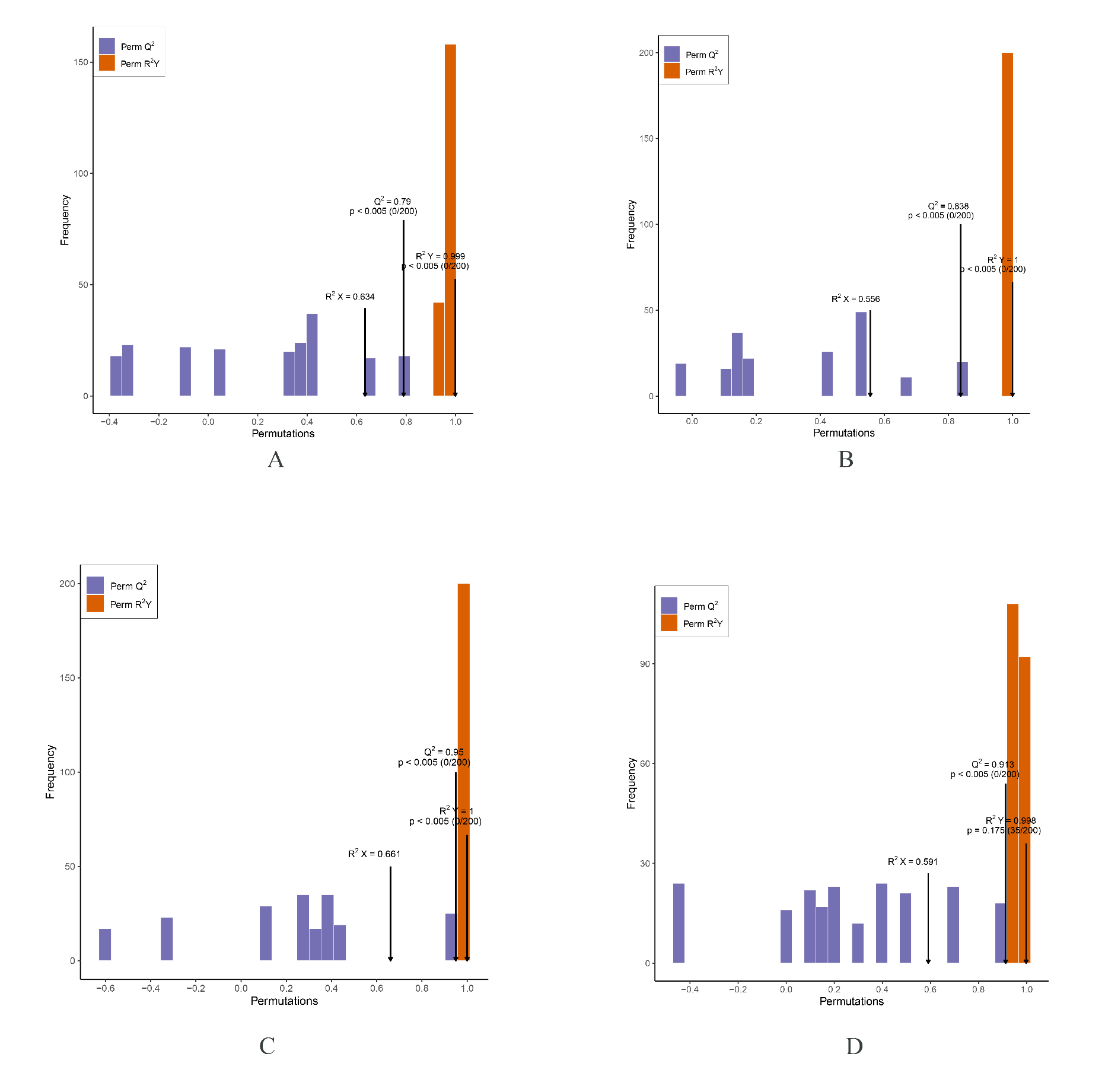


**Supplementary Figure 1.** The OPLS-DA model verification diagram: **A**.CSS vs DXSK; **B**. DMZP vs DXSK; **C**. JMX vs DXSK; **D**. SPK vs DXSK (The horizontal axis represents the accuracy of the model, while the vertical axis represents the frequency of model classification effects; R^2^Y is the interpretation rate of the model to the Y matrix (Class label) and Q^2^ the predictive ability of the model. The model is valid when Q2 > 0.5.)

## Supplementary Tables

**Supplementary Table 1.** Number of metabolites that had significantly different contents in DSXK, CSS, DMZP, JMX, and SPK groups (Up/Down represents the changed trend of the metabolite content in the comparison between the latter group and the former one).

| group | CSS vs DXSK | | DMZP vs DXSK | | JMX vs DXSK | | SPK vs DXSK | |
| --- | --- | --- | --- | --- | --- | --- | --- | --- |
|  | Up | Down | Up | Down | Up | Down | Up | Down |
| flavonoids | 33 | 17 | 40 | 15 | 55 | 7 | 51 | 12 |
| phenolic acids | 23 | 13 | 20 | 9 | 33 | 16 | 26 | 14 |
| alkaloids | 2 | 11 | 11 | 5 | 12 | 18 | 6 | 14 |
| quinones | 1 | 3 | 7 | 1 | 7 | 1 | 5 | 0 |
| lignans and coumarins | 3 | 3 | 2 | 11 | 5 | 6 | 3 | 11 |
| tannins | 0 | 0 | 1 | 0 | 0 | 1 | 0 | 0 |
| terpenoids | 2 | 1 | 0 | 4 | 1 | 6 | 3 | 6 |
| amino acids and their derivatives | 3 | 12 | 4 | 6 | 5 | 39 | 8 | 21 |
| nucleotides and their derivatives | 1 | 0 | 7 | 3 | 3 | 16 | 4 | 7 |
| organic acids | 3 | 6 | 6 | 3 | 4 | 12 | 3 | 8 |
| lipids | 4 | 19 | 5 | 14 | 5 | 43 | 6 | 39 |
| saccharides and alcohols | 5 | 7 | 7 | 3 | 16 | 2 | 2 | 1 |
| vitamins | 0 | 2 | 0 | 2 | 3 | 1 | 1 | 0 |
| steroids | 0 | 0 | 0 | 0 | 0 | 0 | 0 | 0 |
| other compounds | 1 | 9 | 2 | 2 | 5 | 3 | 0 | 5 |
| total | 81 | 103 | 112 | 78 | 154 | 171 | 118 | 138 |

**Supplementary Table 2.** The information of 16 metabolites.

| Index | Molecular Weight (Da) | Formula | Ionization model | Compounds | Class I | Class II |
| --- | --- | --- | --- | --- | --- | --- |
| pmp000344 | 270.053 | C15H10O5 | [M+H]+ | 3',4',7-Trihydroxyflavone | Flavonoids | Flavonoid |
| Zmhn001358 | 300.084 | C13H16O8 | [M-H]- | 4-O-Glucosyl-4-hydroxybenzoic acid | Phenolic acids | Phenolic acids |
| pmn001420 | 342.095 | C15H18O9 | [M-H]- | 1-O-[(E)-Caffeoyl]-D-glucose | Phenolic acids | Phenolic acids |
| Zmhn001793 | 342.096 | C15H18O9 | [M-H]- | 6-O-Caffeoyl-D-glucose | Phenolic acids | Phenolic acids |
| mws0011 | 372.142 | C17H24O9 | [M-H]- | Syringin | Phenolic acids | Phenolic acids |
| pme2459 | 448.101 | C21H20O11 | [M+H]+ | Luteolin-7-O-glucoside (Cynaroside) | Flavonoids | Flavonoid |
| HJN041 | 452.131 | C21H24O11 | [M-H]- | Epicatechin glucoside | Flavonoids | Flavanols |
| pmp000579 | 462.116 | C22H22O11 | [M+H]+ | Diosmetin-7-O-galactoside* | Flavonoids | Flavonoid |
| Hmcp002207 | 478.111 | C22H22O12 | [M+H]+ | Isorhamnetin-7-O-glucoside (Brassicin)* | Flavonoids | Flavonols |
| Lmsn004322 | 504.127 | C24H24O12 | [M-H]- | 1,6-Di-O-caffeoyl-β-D-glucose | Phenolic acids | Phenolic acids |
| pmb3000 | 504.127 | C24H24O12 | [M-H]- | Chrysoeriol-7-O-(6''-acetyl)glucoside | Flavonoids | Flavonoid |
| pmn001672 | 548.174 | C23H32O15 | [M-H]- | Furanofructosyl-α-D-(3-mustard acyl)glucoside | Phenolic acids | Phenolic acids |
| Lmsn003628 | 548.174 | C23H32O15 | [M-H]- | 6′-O-Sinapoylsucrose | Phenolic acids | Phenolic acids |
| pmb2586 | 594.137 | C30H26O13 | [M-H]- | Gallocatechin-(4α→8)-catechin | Flavonoids | Flavanols |
| Lmjp002461 | 610.153 | C27H30O16 | [M+H]+ | Quercetin-3-O-neohesperidoside | Flavonoids | Flavonols |
| pmn001583 | 610.153 | C27H30O16 | [M-H]- | Quercetin-3-O-robinobioside | Flavonoids | Flavonols |
